# Unusual causes of peritonitis in a peritoneal dialysis patient: Alcaligenes faecalis and Pantoea agglomerans

**DOI:** 10.1186/1476-0711-10-12

**Published:** 2011-04-10

**Authors:** Arzu Kahveci, Ebru Asicioglu, Elif Tigen, Elif Ari, Hakki Arikan, Zekaver Odabasi, Cetin Ozener

**Affiliations:** 1 Marmara University School of Medicine, Department of Internal Medicine, Division of Nephrology, Istanbul, Turkey; 2 Marmara University School of Medicine, Department of Infectious Diseases, Istanbul, Turkey

## Abstract

An 87 -year-old female who was undergoing peritoneal dialysis presented with peritonitis caused by Alcaligenes faecalis and Pantoea agglomerans in consecutive years. With the following report we discuss the importance of these unusual microorganisms in peritoneal dialysis patients.

## Case report

The most common pathogens causing peritonitis in peritoneal dialysis (PD) patients are gram positive organisms, Staphylococcus epidermidis and Staphylococcus aureus. Gram negative organisms and fungi are less common causes. Environmental organisms are rarely implicated in patients with peritonitis. With the following report we describe two peritonitis episodes in successive years caused by two unusual microorganism: Alcaligenes faecalis and Pantoea agglomerans.

**Figure 1:**
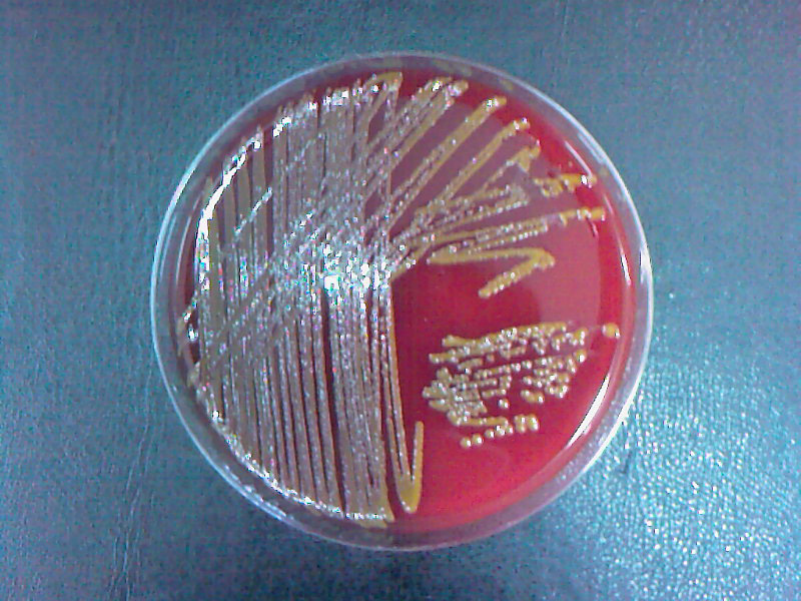
**Pantoea agglomerans strain which produces yellow pigmentation on blood agar**.

A 89-year-old female patient receiving automated peritoneal dialysis presented with abdominal pain and cloudy dialysis fluid. She had a history of diabetes mellitus and had been maintained on APD because of diabetic nephropathy for 10 years. In 2004 she had culture negative peritonitis which was treated with sefazoline and ciprofloxacin. In January 2007, she presented with abdominal pain, cloudy dialysate, and vomiting. Analysis of peritoneal effluent demonstrated white blood cell (WBC) of 700/mm^3^. Fifty mL of peritoneal fluid was centrifugated at 3000 g for 15 minutes, then resuspension of the sediment in 3-5 mL of steril salin inoculated in chocolate-agar, blood-agar and MacConkey agar in microbiology unit. Intraperitoneal sefuroksim (750 mg q12h) and oral ciprofloxacin (500 mg q12h) were given empirically. On the fifth day of treatment a gram-negative, oxidase-positive bacteria with peritrichous flagella, Alcaligenes faecalis was isolated from MacConkey agar. The antibiotic treatment was continued for 21 days and she recovered completely. On February 2009, she presented with cloudy dialysate and abdominal pain. On physical examination, blood pressure was 60/40 mmHg, pulse rate was 96/min, body temperature was 36.5°C. Cardiopulmonary examination was normal. Her abdomen was distended and tender, with normal bowel sounds. Laboratory data showed a blood WBC of 14000/mm^3^. Analysis of peritoneal fluid demonstrated WBC 2700/mm^3^. She was treated with intraperitoneal sefuroksim (750 mg q12 h) and ciprofloxacin (200 mg q12 h) empirically after peritoneal fluid culture sampling. On third day of treatment peritoneal fluid WBC increased to 3400/mm^3^, abdominal pain persisted and the dialysis fluid cultures grew Gram negative rods for which imipenem was started. On blood and chocolate agar plates, the gram-negative rods displayed yellow pigmentation (Figure 1). Oxidase was negative and catalase was positive. The strain was identified as Pantoea agglomerans. The patient's history was not remarkable regarding the possible plant associated injury. On the sixth day, she refused to undergo removal of her PD catheter. Even though appropriate antibiotic therapy was administrated, she died due to septic shock.

## Discussion

Alcaligenes faecalis and Pantoea agglomerans are classified as environmental bacteria and plant pathogens and are rarely reported as being responsible for clinically significant infections in human. Alcaligenes faecalis is a gram negative oxidase positive rod with peritrichous flagella which exists in soil and water. It is also found in the alimentary tract as a harmless saprophyte in 5-19% of the human population. Systemic infection with this organism is very uncommon. To our knowledge, this is the second report of a PD patient with Alcaligenes faecalis peritonitis. This patient recovered by antimicrobial therapy alone and did not require catheter removal similar to previous patient reported by Kavuncuoglu et al. [1]. However another species of Alcaligenes, Alcaligenes xylosoxidans has been reported to cause peritonitis in PD patients previously [2]. The majority of these patients required catheter removal because of resistance to most antibiotic therapies. Even though Alcaligenes faecalis is presumed to be resistant to antibiotics, as in our isolate, our case emphasizes the importance of sensitivity testing when it is isolated.

Pantoea agglomerans is another gram negative aerobic bacillus which caused peritonitis in our patient in 2009. It can be isolated from plants, soil and feculent material. Pantoea species are rare causes of clinically significant infection similar to A. Faecalis. However in recent years, an increased rate of Pantoea peritonitis in PD patients has been reported (Table 1) [3-9]. Plant associated injury is the most commonly attributed aetiology. However it has been linked to contaminated blood products, intravenous fluids, total parenteral nutrition and anesthetic agents. Furthermore oral contamination or bacterial translocation from gastrointestinal system may also be responsible for Pantoea infections. Peritoneal dialysis catheters are not known to be a risk factor of infection by Pantoea [5]. Regarding the previous Alcaligenes faecalis peritonitis in our patient we believe that plant associated injury or bacterial translocation may be responsible factors for the peritonitis episodes.

**Table 1:** Cases of Peritoneal Dialysis (PD) Associated Peritonitis with Pantoae Agglomerans

Case (Ref.)	Type of PD	Sex/Age	Proposed etiology	Antbiotics used/route	Outcome
1 [3]	NA	F/2	Teeting to catheter	Cefotaxime, Gentamisin/IP	Cured, catheter replaced new one

2 [4]	CAPD	F/49	Rose-thorn injury	Ceftazidime, Amikasin/IP	Cured

3 [5]	CCPD	M/65	Unknown	Ciprofloxacin/IV	Cured

4 [6]	CAPD	M/51	Rose-thorn injury	Cefepime/IP	Cured

5 [7]	NA	F/52	Unknown	Ciprofloxacin/oral	Cured

6 [8]	CAPD	M/45	Thorn injury	Ciprofloxacin/IP	Cured

7 [9]	CCPD	M/56	Unknown	Tobramycin/NA	Cured

Our case	APD	F/89	Unknown	İmipenem/IV	Patient died

NA, not adjusted; CAPD, Continuous Ambulatory Peritoneal Dialysis; CCPD, Continuous Cycling Peritoneal Daialysis; APD, Automated Peritoneal Dialysis; IP, Intraperitoneal; IV, Intravenous

In conclusion, although these organisms are rarely fatal and are commonly considered to be contaminants they can cause symptomatic peritonitis, which in turn leads to death. It is therefore very important to view these organisms as pathogens rather than contaminants when isolated in patients with peritonitis. Microbiological antibiotic testing should always be requested from the laboratory and patients should be treated accordingly. When there is no improvement with antibiotic therapy, early removal of the peritoneal catheter should be planned since these infections may prove fatal.

## Consent

Written informed consent was obtained from the patient's daughter for publication of this case report and accompanying images. A copy of the written consent is available for review by the Editor-in-Chief of this journal.

## Competing interests

The authors declare that they have no competing interests.

## Authors' contributions

AK, EA, ET and EA made substantial contributions to the design, and the acquisition and interpretation of data. HA, ZO and CO revised the manuscript critically for important intellectual content. All authors read and approved the final manuscript.
